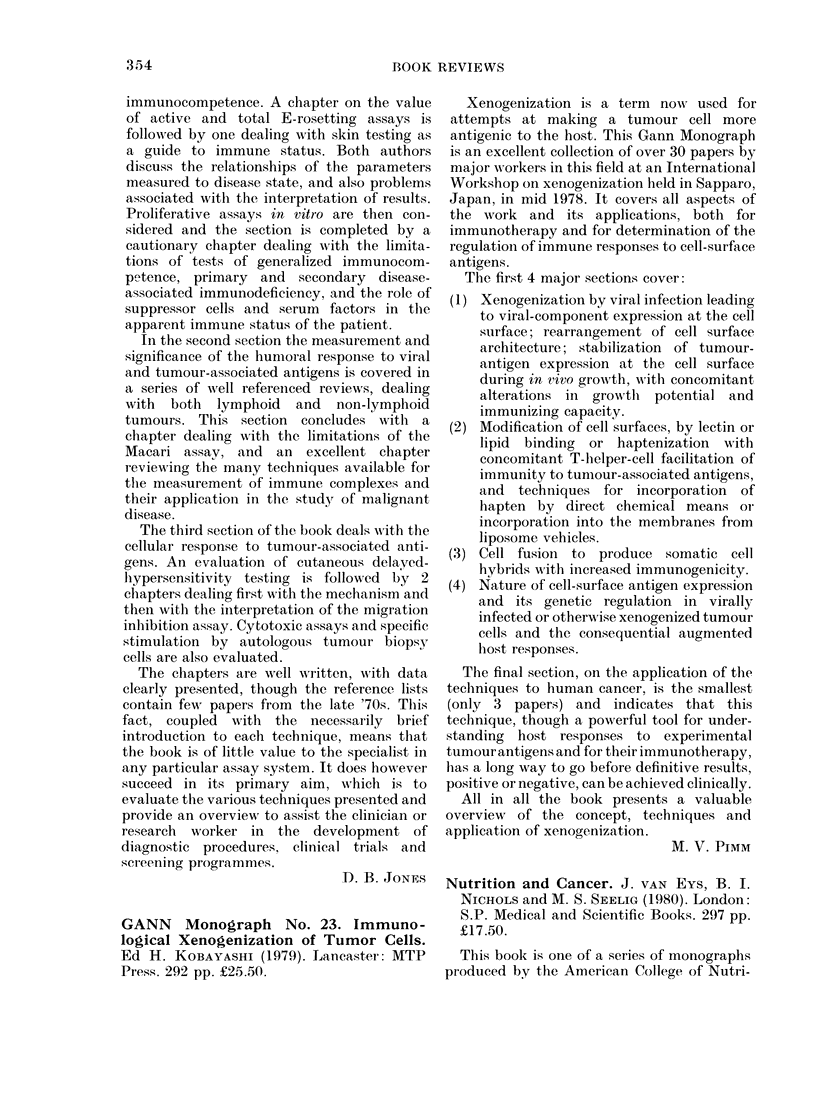# GANN Monograph No. 23. Immunological Xenogenization of Tumor Cells

**Published:** 1980-08

**Authors:** M. V. Pimm


**GANN Monograph No. 23. Immunological Xenogenization of Tumor Cells.** Ed H. Kobayashi (1979). Lancaster: MTP Press. 292 pp. £25.50.

Xenogenization is a term now used for attempts at making a tumour cell more antigenic to the host. This Gann Monograph is an excellent collection of over 30 papers by major workers in this field at an International Workshop on xenogenization held in Sapparo, Japan, in mid 1978. It covers all aspects of the work and its applications, both for immunotherapy and for determination of the regulation of immune responses to cell-surface antigens.

The first 4 major sections cover:

(1) Xenogenization by viral infection leading to viral-component expression at the cell surface; rearrangement of cell surface architecture; stabilization of tumour-antigen expression at the cell surface during *in vivo* growth, with concomitant alterations in growth potential and immunizing capacity.
(2) Modification of cell surfaces, by lectin or lipid binding or haptenization with concomitant T-helper-cell facilitation of immunity to tumour-associated antigens, and techniques for incorporation of hapten by direct chemical means or incorporation into the membranes from liposome vehicles.
(3) Cell fusion to produce somatic cell hybrids with increased immunogenicity.
(4) Nature of cell-surface antigen expression and its genetic regulation in virally infected or otherwise xenogenized tumour cells and the consequential augmented host responses.

The final section, on the application of the techniques to human cancer, is the smallest (only 3 papers) and indicates that this technique, though a powerful tool for understanding host responses to experimental tumour antigens and for their immunotherapy, has a long way to go before definitive results, positive or negative, can be achieved clinically.

All in all the book presents a valuable overview of the concept, techniques and application of xenogenization.

M. V. Pimm